# A new definitive host for *Moniliformis cestodiformis* (Acanthocephala: Moniliformidae): first report of a naturally infected European hedgehog (*Erinaceus europaeus*)

**DOI:** 10.1590/S1984-29612023014

**Published:** 2023-03-17

**Authors:** David Wilson Ramilo, João Tomás Cruz, Omar Amin, Carolina Fragoso, Erica Brazio, Jorge Correia, Luís Cardoso, Isabel Pereira da Fonseca

**Affiliations:** 1 Faculdade de Medicina Veterinária, Universidade Lusófona – Centro Universitário de Lisboa, Lisboa, Portugal; 2 Centro de Investigação Interdisciplinar em Sanidade Animal – CIISA, Faculdade de Medicina Veterinária, Universidade de Lisboa – ULisboa, Lisboa, Portugal; 3 Laboratório Associado para a Ciência Animal e Veterinária – AL4AnimalS, Portugal; 4 Institute of Parasitic Diseases, Parasitology Center, Inc. – PCI, Arizona, United States of America; 5 Centro de Reabilitação de Animais Selvagens – LxCras, Lisboa, Portugal; 6 Departamento de Ciências Veterinárias, Centro de Ciência Animal e Veterinária – CECAV, Universidade de Trás-os-Montes e Alto Douro – UTAD, Vila Real, Portugal

**Keywords:** Acanthocephala, Erinaceus europaeus, Moniliformis cestodiformis, Acanthocephala, Erinaceus europaeus, Moniliformis cestodiformis

## Abstract

European hedgehogs, *Erinaceus europaeus* (Linnaeus, 1758), are small mammals found in western Europe and also in parts of northern Europe. They can be seen in rural, suburban and urban areas, but are usually found in grassland with edge habitats. These animals are omnivorous and serve as definitive or paratenic hosts for several parasites, including acanthocephalans (phylum Acanthocephala). During necropsy of a European hedgehog, a single adult parasite was collected from the intestinal lumen and preserved in 70% ethanol. After morphological evaluation of the specimen, it was identified as *Moniliformis cestodiformis* (von Linstow, 1904) (Acanthocephala: Moniliformidae). This is the first report of *M. cestodiformis* in a European hedgehog, as well as in Europe. More epidemiological studies need to be carried out to map the location and prevalence of this parasite in Portugal and the European continent.

## Resumo

Os ouriços, *Erinaceus europaeus* (Linnaeus, 1758), são pequenos mamíferos que se localizam na Europa ocidental e também em regiões do Norte da Europa. Eles podem ser avistados em áreas rurais, suburbanas e urbanas, mas são geralmente encontrados no campo no limite daqueles hábitats. São animais omnívoros e servem de hospedeiros definitivos ou paratênicos de muitos parasitas, incluindo acantocéfalos (filo Acanthocephala). Durante a necropsia de um ouriço, um exemplar adulto acantocéfalo foi recolhido do lúmen intestinal e preservado em etanol a 70%. Depois de uma avaliação morfológica desse exemplar, este foi identificado como *Moniliformis cestodiformis* (von Linstow, 1904) (Acanthocephala: Moniliformidae). Esta é a primeira referência de *M. cestodiformis* num ouriço, assim como na Europa. Mais estudos epidemiológicos necessitam ser realizados, para localizar este parasita e calcular a sua prevalência em Portugal e no continente europeu.

## Introduction

European hedgehogs, *Erinaceus europaeus* (Linnaeus, 1758) are small nocturnal mammals native to western and northern Europe (Reeve, 1994; Pfäffle, 2011; Sangster et al., 2016; CABI, 2022). They can be seen in rural, suburban and urban areas, but are usually found in grassland with edge habitats (Jahfari et al., 2017). This kind of habitat preferences often results in direct or indirect contact with wildlife, domestic animals and also humans (Amori, 2016). European hedgehogs are omnivorous, feeding mainly on invertebrates including beetles, caterpillars, woodlice and other insects, as well as snails, slugs and earthworms. Hedgehogs can also eat vertebrates, such as snakes, vipers, frogs, toads, fish, birds and their eggs, and small mammals (Pfäffle, 2011; Naem et al., 2015). Because of these food preferences, *E. europaeus* may act as a definitive or paratenic host of several parasites, some of them being zoonotic, like *Trichinella* spp. and *Leptospira* spp. (Jones et al., 2005; Riley & Chomel, 2005; Pozio, 2007; CABI, 2022). Furthermore, several parasites can affect *E. europaeus*, like protozoa, fleas, mites, ticks and helminths, including acanthocephalans (Pfäffle, 2011).

The genus *Moniliformis* (Moniliformida: Moniliformidae) Travassos, 1915 includes 20 recognized species (Amin, 2013; Amin et al., 2016; Gomes et al., 2020; Lynggaard et al., 2021; Dai et al., 2022). The adult forms are medium-sized thorny-headed worms with a very small proboscis when compared to their trunk. The worms are pseudosegmented rounded anteriorly and posteriorly (Golvan, 1962; Amin et al., 2016).

*Moniliformis* spp. infect warm-blooded vertebrates, including mammals and birds. For example, *M. moniliformis* uses rodents and humans as definitive hosts, being zoonotic in countries where insects, such as cockroaches, are eaten raw (Coomansingh-Springer et al., 2019). Five *Moniliformis* spp. are known to infect hedgehogs, but only *Moniliformis cestodiformis* (von Linstow, 1904) Travassos, 1917 has been reported in *Erinaceus* spp. (Amin et al., 2016).

Knowledge about the parasites of *E. europaeus* from mainland Portugal is scarce. Concerning *M. cestodiformis*, it has only been reported in *Erinaceus* spp. in West Africa in 1925 (Amin et al., 2016). The present study describes for the first time *M. cestodiformis* in an *E. europaeus* specimen and also represents the first report of this parasite in Europe.

## Material and Methods

In May 2019, a female European hedgehog in poor physical condition, found in Monsanto forest park, within the municipality of Lisbon, was brought to the Wild Animal Rehabilitation Centre of Lisbon (LxCRAS), together with five offspring. The female stayed with the pups during the first 9 days but, due to her refusal to eat, they were separated. She was treated with natural complementary food (Anima-Strath^Ⓡ^, 2.5 ml, *per os* [PO]), a nutritional complement (Duphalyte^Ⓡ^, 12 ml/kg, PO) and 3 ml of saline solution subcutaneously (SC), at body temperature. On the next day, the female hedgehog was treated with fenbendazole (Panacur^Ⓡ^, 100 mg/kg, PO). On the 11^th^ day the animal was warmed up due to hypothermia, but did not survive.

During necropsy, a single adult parasite was collected from the intestinal lumen and preserved in 70% ethanol. The parasite was measured and some eggs were collected. The obtained material was prepared on a slide with Hoyer’s medium and observed under an optical microscope. To identify the parasite specimen, an identification key was used (Amin et al., 2016). Information regarding proboscis hooks and their roots, the female reproductive system and gonopore were collected. To confirm the species, proboscis, proboscis receptacle, lemnisci, hooks and its roots, the terminal part of reproductive system and eggs were observed and measured.

## Results

**Figure 1:**
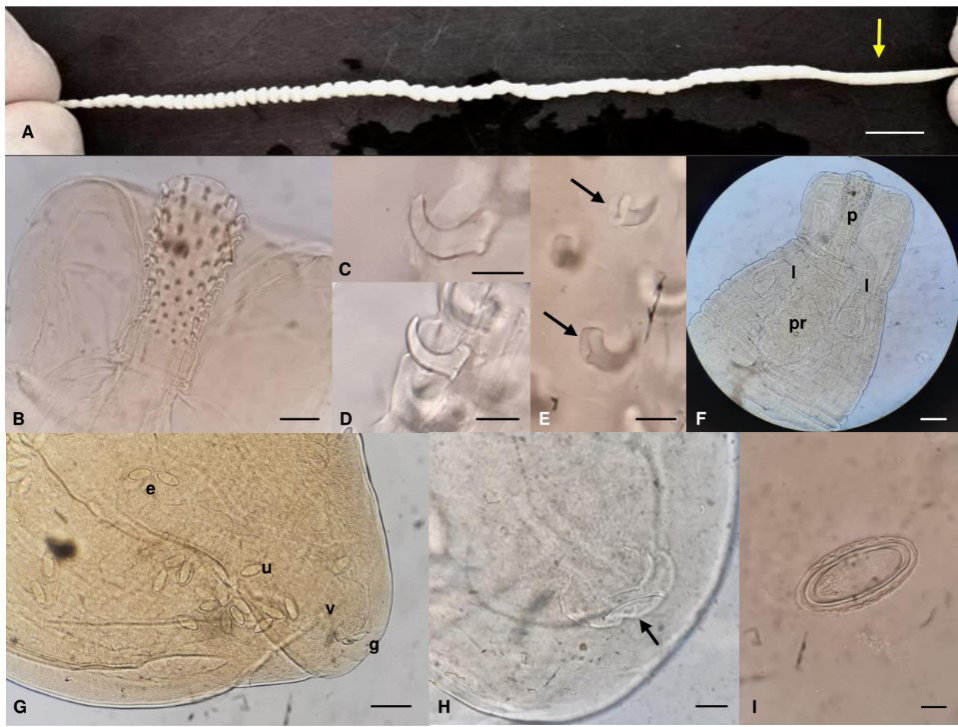
*Moniliformis cestodiformis.***A:** Adult female specimen. Morphological conformation, with pseudosegmentation of trunk (yellow arrow). **B:** Proboscis. **C-D:** Hooks. **E:** Hook roots (black arrows) with an invagination in the ventral middle part. **F:** Anterior part of the body showing proboscis (p), proboscis receptacle (pr) and lemnisci (l); the diagonal musculature of the proboscis receptacle is also visible. **G:** Posterior part of the body showing the eggs (e), uterus (u), vagina (v) and gonopore (g). **H:** Gonopore and its orifice (black arrow). **I:** Egg. Scale bar on A: 1 cm. Scale bar on B: 100 µm. Scale bar on C-E and I: 20 µm. Scale bar on F and G: 200 µm. Scale bar on H: 40 µm.

The parasite presented a total length of approximately 13 cm (Figure 1A). Due to its morphological conformation, being round anteriorly and posteriorly and pseudosegmented in-between (Figure 1A), it was identified as belonging to genus *Moniliformis*.

Except where millimeters (mm) are indicated, the following measurements are all in micrometers (μm). Proboscis was 475 long and 195 wide (Figure 1B); it had 16 rows of six to 10 hooks each. Hook blades decreased in size posteriorly. The largest hooks had 23.41 by 9.6 and the smallest 4.78 by 2.06 (Figures 11D); hook roots were stout and inserted in the ventral middle part (Figure 1E); proboscis receptacle was 1.13 mm long and 396 wide (Figure 1F). Lemnisci was 1.68 mm long and 166 wide posteriorly (Figure 1F).

Regarding the reproductive system, uterus was 640.5 long, and vagina 135.8 long and 32.5 wide (Figure 1G). Gonopore was nearly terminal (Figure 1G) and its orifice was 67.7 long in its major axis (Figure 1H). Eggs were 87.5-95 long by 45-50 wide (Figure 1I). Likewise, all measurements are in micrometers (μm). Accordingly, the female specimen was identified as *Moniliformis cestodiformis* (von Linstow, 1904).

## Discussion

Parasites are known to have a substantial impact on population dynamics of their hosts (Irvine, 2006). They are a threat to debilitated hedgehogs, frequently leading to morbidity and even death. Some parasites can also represent a zoonotic risk and pose a possible cross infection with pets (Wright, 2014). The hedgehog described in this study was in a poor physical condition and the presence of *M. cestodiformis* may have impaired its health improvement.

Although several acanthocephalan species have been reported in hedgehogs (Pfäffle, 2011), only a few earlier publications mention *M. cestodiformis*. This species was originally described in 1904 by von Linstow as *Echinorhynchus cestodiformis* from two different African species of hedgehogs, *Atelerix albventris* and *Atelerix frontalis* (Travassos, 1917). Approximately a century ago, Travassos (1917) transferred it from the genus *Echinorhynchus* to the genus *Moniliformis* (Travassos, 1915). Southwell & Macfie raised *Moniliformis erinacei* as a new species from measurements obtained from a male and a female specimen in *Erinaceus* spp. from West Africa (Sandground, 1926). According to Amin et al. (2016), these two species, *M. cestodiformis* and *M. erinacei*, are the same and the latter must not be considered as valid species.

None of the previous parasitological surveys in European hedgehogs between 1926 and 2016, e.g., Pfäffle et al. (2014), refers to *M. cestodiformis*. One paper (Amin et al., 2016) includes this species in identification keys and only refers to *M. erinacei* in *Erinaceus* spp. from West Africa. The geographical distribution of this parasite is probably not wide enough to be accounted for during hedgehog necropsies or coprological analysis of their feces, as it happens with some other parasites, such as trematode *Brachylecithum mackoi* in European hedgehogs from Elba island (Casanova & Ribas, 2004).

## Conclusion

The present study is a major contribution to the knowledge of the European hedgehog parasitological fauna from Portugal and Europe, providing valuable data concerning *M. cestodiformis* and bringing to light the first reference in *E. europaeus* from Portugal and the European continent. More collections and epidemiological studies must be performed to understand its prevalence and localization in Portugal and also in Europe, since work concerning *M. cestodiformis* is non-existent according to the best of our knowledge.

## References

[B001] Amin OM, Heckmann RA, Osama M, Evans RP (2016). Morphological and molecular descriptions of *Moniliformis saudi* sp. n. (Acanthocephala: Moniliformidae) from the desert hedgehog, *Paraechinus aethiopicus* (Ehrenberg) in Saudi Arabia, with a key to species and notes on histopathology. Folia Parasitol (Praha).

[B002] Amin OM (2013). Classification of the Acanthocephala. Folia Parasitol (Praha).

[B003] Amori G (2016). Erinaceus europaeus. The IUCN Red list of threatened species.

[B004] CABI (2022). Invasive species compendium: Erinaceus europaeus (European hedgehog).

[B005] Casanova JC, Ribas A (2004). Description of *Brachylecithum mackoi* n. sp. (Digenea: Dicrocoeliidae) from the European hedgehog, *Erinaceus europaeus* (Insectivora: Erinaceidae). J Parasitol.

[B006] Coomansingh-Springer C, Vishakha V, Acuna AM, Armstrong E, Sharma RN (2019). Internal parasitic burdens in brown rats (*Rattus norvegicus*) from Grenada, West Indies. Heliyon.

[B007] Dai G-D, Yan H-B, Li L, Zhang L-S, Liu Z-L, Gao S-Z (2022). Molecular characterization of a new *Moniliformis* sp. from a plateau zokor (*Eospalax fontanierii baileyi*) in China. Front Microbiol.

[B008] Golvan Y-J (1962). Le phylum des *Acanthocephala*. IV. La classe des *Archiacanthocephala* (A. Meyer 1931). Ann Parasitol Hum Comp.

[B009] Gomes APN, Costa NA, Gentile R, Vilela RV, Maldonado A (2020). Morphological and genetic description of *Moniliformis necromysi* sp. n. (Archiacanthocephala) from the wild rodent *Necromys lasiurus* (Cricetidae: Sigmondontinae) in Brazil. J Helminthol.

[B010] Irvine RJ (2006). Parasites and the dynamics of wild animal populations. Anim Sci.

[B011] Jahfari S, Ruyts SC, Frazer-Mendelewska E, Jaarsma R, Verheyen K, Sprong H (2017). Melting pot of tick-borne zoonoses: the European hedgehog contributes to the maintenance of various tick-borne disease in natural cycles urban and suburban areas. Parasit Vectors.

[B012] Jones C, Moss K, Sanders M (2005). Diet of hedgehogs (*Erinaceus europaeus*) in the upper Waitiki Basin, New Zealand: implications for conservation. N Z J Ecol.

[B013] Lynggaard C, García-Prieto L, Guzmán-Cornejo C, García-Varela M (2021). Description of a new species of *Moniliformis* (Acanthocephala: Moniliformidae) from *Peromyscus hylocetes* (Rodentia: Cricetidae) in Mexico. Parasitol Int.

[B014] Naem S, Pourreza B, Gorgani-Firouzjaee T (2015). The European hedgehog (*Erinaceus europaeus*), as a reservoir for helminth parasites in Iran. Vet Res Forum.

[B015] Pfäffle M, Černá Bolfíková B, Hulva P, Petney T (2014). Different parasite faunas in sympatric populations of sister hedgehog species in a secondary contact zone. PLoS One.

[B016] Pfäffle MP (2011). Influence of parasites on fitness parameters of the European hedgehog (Erinaceus europaeus).

[B017] Pozio E (2007). World distribution of *Trichinella* spp. infection in animals and humans. Vet Parasitol.

[B018] Reeve N (1994). Hedgehogs..

[B019] Riley PY, Chomel BB (2005). Hedgehog zoonoses. Emerg Infect Dis.

[B020] Sandground JH (1926). On an unusual occurrence of *Moniliformis moniliformis* (Acanthocephala) as a parasite of toads and lizards in Central America. Trans Am Microsc Soc.

[B021] Sangster L, Blake DP, Robinson G, Hopkins TC, Sa RCC, Cunningham AA (2016). Detection and molecular characterisation of *Cryptosporidium parvum* in British European hedgehogs (*Erinaceus europaeus*). Vet Parasitol.

[B022] Travassos L (1917). Contribuições para o conhecimento da fauna helmintolojica brazileira. Mem Inst Oswaldo Cruz.

[B023] Wright I (2014). Parasites affecting wild European hedgehogs: disease potential and zoonoses. Companion Anim.

